# New logarithm-based discrimination formula for differentiating thalassemia trait from iron deficiency anemia in pregnancy

**DOI:** 10.1186/s12884-023-05365-3

**Published:** 2023-02-08

**Authors:** Xiao Shuang, Wang Zhenming, Mei Zhu, Sun Si, Li Zuo

**Affiliations:** 1 School of Economics and Business Administration, Chongqing University, Chongqing, 400044 China; 2 Department of Medicine and Education, People’s Hospital of Chongqing Liang Jiang New Area, Chongqing, 401121 China; 3 Department of Obstetrics, People’s Hospital of Chongqing Liang Jiang New Area, Chongqing, 401121 China; 4 Department of Medical Laboratory, People’s Hospital of Chongqing Liang Jiang New Area, Chongqing, 401121 China

**Keywords:** Blood, Thalassemia trait, Iron deficiency anemia, Discrimination, Diagnostic performance

## Abstract

**Background:**

Thalassemia trait (TT) and iron deficiency anemia (IDA) are the most common conditions of microcytic hypochromic anemia (MHA) in pregnant women. Accurate discrimination between TT and IDA is an important issue, and better methods are urgently needed. Although considerable RBC formulas and indices have been developed since 1973, distinguishing between IDA and TT is still a challenging problem due to the diversity of various anemic populations. To address this problem, we assessed the diagnostic function of 43 different differential formulas in patients with microcytic anemia by using accuracy measures and recommending a new log-based differential formula.

**Methods:**

The data of 430 pregnant women (229 with TT and 201 with IDA) were enrolled, and 44 formula performances were evaluated with receiver operating characteristic (ROC) analysis.

**Results:**

The newly introduced logarithm-based formula XS-1 performs better than the general discriminant index with sensitivity and specificity of 82.10 and 89.05, which are better than other formulas. In the pregnant population, the Shine and Lal and Roth..SVM. formulas have shown excellent performance, while other formulas showed poorer discriminative abilities in our study than in the original authors.

**Conclusion:**

The logarithm-based formula XS-1 can be used to screen thalassemia and iron deficiency anemia during the first trimester. Considering the particularity of pregnancy, medical personnel in different regions should choose a screening formula similar to that of the local region and population when identifying thalassemia in pregnancy. Any formula should be independently verified locally before use. For the convenience of the health care team and experimental scientists, a web-based tool has been established at http://yyy.yiyiy.top/XS-1/ by which users can easily get their desired screening test result without going through the underlying mathematical and computational details.

## Introduction

Anemia is a global problem that has a serious impact on mothers and infants, and severe anemia is an independent factor for maternal mortality, with a mortality rate of 2.36% [1], while the rate of anemia in women during pregnancy is as high as 41.8% [2]. Although Thalassemia(TT) is not as high as Iron Deficiency Anemia (IDA) in incidence, it is also a kind of Microcytic hypochromic anemia (MHA), which can be easily confused with IDA for the similar symptoms and signs. Thalassemia is a monogenic disease caused by the reduction or inhibition of hemoglobin chain synthesis [3], while IDA is caused by iron deficiency. The causes of the two anemia are different, hence need different treatments. Doctors need to accurately determine the type of anemia of pregnant women and give medical orders to take iron supplements or not. Furthermore, although the vast majority of pregnant women with TT are asymptomatic and usually not associated with any clinical disease, early detection can prevent the birth of a child with homozygous thalassemia syndrome.

Thalassemia is so called because it was first discovered among peoples around the Mediterranean Sea. Thalassemia is mainly distributed in the tropical and subtropical areas of high global malaria rate, including the south of the Yangtze River of China. The carrying rate of the thalassemia gene is 16.83%-17.83% in Guangdong Province and 24.51% in Guangxi [4, 5]. In the first 3 months of pregnancy, seven provinces and cities in China, including Chongqing, recommend thalassemia screening as a mandatory item for antenatal care [6].

Whole blood cell testing is the preliminary means for judging anemia, and serum ferritin and genetic testing are required to determine its type. However, these tests are complex and expensive, require professional testing institutions, thus cannot be used as routine inspection items, and are more difficult in poor and remote areas. Thalassemia is incurable, and a routine iron supplement may cause adverse reactions of iron overload, such as gestational diabetes [7], placental abnormalities, etc. [8]. Studies have found that the maternal β-thalassemia gene independently correlates with hematological diseases in offspring. Identifying thalassemia and iron deficiency anemia in pregnancy is of great significance for treating pregnancy anemia and neonatal health care [9].

In recent decades, RBC index and various formulas derived from it have been widely used to discriminate between IDA [10–21] and thalassemia in adults or children. However, they have not been independently validated in pregnant women, and there is no discriminant formula for pregnant women (Discriminant et al. formula, DF). With limited medical facilities, especially for pregnant women in remote areas, a concise and effective mathematical formula is an effective tool for screening TT in pregnancy. Pregnancy is a special process for the blood of pregnant women will undergo considerable changes. In order to find a suitable identification formula, this study will construct a logarithm-based identification formula XS-1 and compare its performance with the other 43 mathematical formulas published so far with good performance. We used a hospital database containing information on pregnant women with well-defined thalassemia genes to achieve the two goals.

## Materials and methods

### Criteria for selecting the patient groups

We reviewed the profiles of all pregnant women who gave birth at People's Hospital of Chongqing Liang Jiang New Area from January 2018 to December 2021. All 546 pregnant women who received prenatal thalassemia DNA testing were enrolled, in which 32 women aged < 20 years or > 40 years old, 5 delivered twins and multiple births, and 1 had a stillbirth were excluded. Also, 53 cases with thalassemia gene and serum ferritin (SF) < 20 μg/L were excluded, and 25 cases without thalassemia gene and serum ferritin (SF) ≥ 20 μg/L were excluded. Finally, a total of 430 cases were included in this study. This study was reviewed and approved by the Ethics Review Committee of People's Hospital of Chongqing Liang Jiang New Area (No. LLYY-LL2022-037), and all methods were performed following relevant guidelines and regulations.

### Definition and outcomes

Routine blood data was obtained from pregnant women in the first trimester (0–12 weeks), fasting venous blood for about 3 mL. The full blood count was completed by Sysmex XN-1500 automatic blood analyzer in the Laboratory Department of the First People's Hospital of Chongqing Liang Jiang New Area, the evaluation included: WBC, RBC, Hb, HCT, MCV, MCH, MCHC, RDW-CV, RDW-SD, and PLT.

The characteristics of thalassemia were determined by DNA detection. 2 mL of fasting venous blood was drawn from pregnant women, from which three common deletion α-thalassemia genes (-SEA, -α3.7, -α4.2) were detected with the gap-PCR amplification method, and eight common sites and nine uncommon sites of β-globin gene mutation were detected by PCR-membrane reverse dot hybridization technique. The detection instrument is Eppendorf Mastercycler Gradient. The parallel joint analysis was used: any positive result of the two indicators is determined positive.

Pregnant women who carried the thalassemia gene and serum ferritin (SF) ≥ 20 μg/L were divided into the study group, and those without the thalassemia gene and serum ferritin (SF) < 20 μg/L in the control group [22].

### Development of the new index

The fission limit to the growth of blood cells principle (Formula 1) was used to detect the index between the two groups with strategies developed by logarithm-based formula with e as the base, with the maximal area under the curve (AUC) displayed by the index as a single parameter to construct a formula.

The red blood cells, as a natural object, has a growth process satisfies the natural growth limit, that is, the growth per unit time can expand at most e times of the natural constant (Formula 1). Pregnant women with thalassemia or iron deficiency anemia have the problem of insufficient hemoglobin or morphological defects, leads to unable to effectively carry oxygen molecules. The body will solve the problem by increasing the number of red blood cells and changing the cell morphology. However, different diseases have different rules of red blood cell growth. Logarithm of Natural logarithm (exp-base) was used to develop several strategies to explore the difference of red cell growth in different diseases, and the maximum area under the differential expression curve (AUC) is taken as a single-parameter construction formula.

1 $${\exp\;=\;\lim}_{n\rightarrow\infty\;}\left(1+\frac{100}n\right)^n=\;2.718281828\dots\;\dots$$

### Statistical analysis

Using SPASS15 to calculate the sample size, 200 positive and negative results, respectively, would provide a power of 80% to detect the difference under the invalidation AUC of 0.85 and validation AUC of 0.9. The receiver operating characteristic (ROC) curve was used for analysis, with the data points exclusively from subjects with thalassemia characteristics. Descriptive data are presented as numbers (percentages). Measure index of the relationship between exposure and result was expressed as odds ratios (ORs) with 95% confidence intervals (CIs). A value of *P* < 0.05 was considered statistically significant. Data analysis was performed using R version 4.1.3. Using epiR of R package to calculate the measure of accuracy and 95% confidence interval( 95% CI). ROC curve analysis was performed using the pROC software package. Also, the Optimal Cutpoints package used the Youden index to calculate new cutoffs of the discriminant formula.

## Results

### Baseline characterizes of participant

There were 229 pregnant women (53.26%) with thalassemia characteristics (TT) and 201 pregnant women (46.74%) with iron deficiency anemia in this study. (Table 1). There was no significant difference in baseline characteristics between the two groups (*p* > 0.05), with balanced baseline and comparable data. The three groups of data are comparable. There were significant differences in hematological parameters between TT and IDA groups (*p* > 0.05).

**Table 1:** Demographic characteristics and hematological parameters of pregnant women (TT and IDA)

Variable	IDA *N* = 201	TT *N* = 229	*P* value
Age^a^	27.0 ± 4.50	28.0 ± 4.60	0.0272
**Nationality**			1
Others	12 (6.0)	14 (6.1)	
Han	189 (94.0)	215 (93.9)	
**Bloot Type**^b^			0.6731
A	71 (35.3)	72 (31.4)	
AB	16 (8.0)	24 (10.5)	
B	45 (22.4)	57 (24.9)	
O	69 (34.3)	76 (33.2)	
**WBC**(× 10^9^/L) ^a^	7.8 ± 1.90	8.3 ± 2.20	0.0184
**RBC**(× 10^12^/L) ^a^	4.1 ± 0.36	4.8 ± 0.54	< 0.0001
**Hb**(g/L) ^a^	120.0 ± 12.00	110.0 ± 14.00	< 0.0001
**HCT**(%)^a^	36.0 ± 3.10	34.0 ± 4.00	< 0.0001
**MCV**(fL) ^a^	87.0 ± 5.50	72.0 ± 9.40	< 0.0001
**MCH**(pg) ^a^	29.0 ± 2.30	23.0 ± 3.40	< 0.0001
**MCHC**(g/L) ^a^	340.0 ± 9.90	320.0 ± 10.00	< 0.0001
**RDW-CV**(%)^a^	14.0 ± 1.90	15.0 ± 1.80	< 0.0001
**RDW-SD**(fL) ^a^	44.0 ± 4.70	39.0 ± 4.40	< 0.0001
**PLT**(× 10^9^/L) ^a^	220.0 ± 52.00	240.0 ± 62.00	0.0219

*P* value were from T-test and Chisquare test respectively

^a^Mean ± SD

^b^N(%)

### ROC of 44 discriminant formulas

**Fig. 1:**
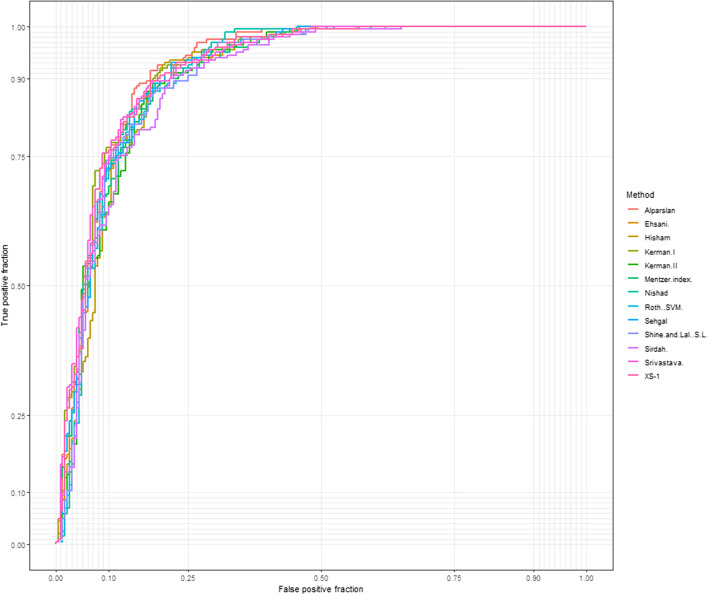
Performance comparisons among 13 discriminant formulas using ROC curves

The discriminant formulas and their cutoff values are detailed in Table 2. Table 3 shows the diagnostic performance of the 44 discriminant formulas, with data of the area under the curve (AUC), accuracy, sensitivity, specificity, and Youden index, ranked according to the years of publication of the formulas. 13 formulas with AUC > 0.9, ranked from high to low: Srivastava, Kerman.I, Alparslan, XS-1, Mentzer, Ehsani., Nishad, Sehgal, S&L, Sirdah., Kerman.II, Hisham, and Roth..SVM (Fig. 1). Among the 13 formulas, those with a sensitivity > 70% are Kerman.II (99.56%), XS-1 (82.10%), S&L (72.93%), and Roth..SVM (72.93%). Except for Kerman.II, whose specificity is 1%, the specificities of the other three formulas are all greater than 85%. The ranking of the Youden index of the four formulas is XS-1 (0.71), S&L (0.66), Roth..SVM ( 0.66), and Kerman.II (0.01).

**Table 2:** 44 discriminant formulas and their cut-offs of thalassemias and iron-deficiency anemias

**No**	**DF** (Reference Publication)	**Calculation**	**Thalassemia Cut-off Value**	**Year of publication(Year)**
1	England and Fraser (E&F) [17]	MCV – RBC – (5 Hb) – 3.4	< 0	1973
2	RBC [17]	RBC	> 5	1973
3	Mentzer [23]	MCV / RBC	< 13	1973
4	Srivastava [24]	MCH / RBC	< 3.8	1973
5	Shine and Lal (S&L) [25]	MCV^2^ × MCH/100	< 1530	1977
6	Bessman [10]	RDW	< 15	1977
7	Ricerca [26]	RDW / RBC	< 3.3	1987
8	Green and King(G&K) [19]	MCV^2^ × RDW / 100 Hb	< 65	1989
9	Das Gupta [20]	1.89RBC-0.33RDW-3.28	> 0	1994
10	Jayabose(RDW index) [27]	(MCV × RDW)/RBC	< 220	1999
11	TI (MCHD) [28]	MCH/MCV	< 0.34	1999 1999
12	TI (MDHL) [28]	(MCH × RBC)/MCV	> 1.5	
13	Huber– Herklotz [29]	(MCH × RDW × 0.1/ RBC) + RDW	< 20	2004
14	Sirdah [30]	MCV – RBC – (3 Hb)	< 27	2007
15	Kerman I [14]	(MCV × MCH)/RBC	< 300	2008 2008
16	Kerman II [14]	(MCV × MCH × 10)/(RBC × MCHC)	< 85	
17	Ehsani [31]	MCV – (10 RBC)	< 15	2008
18	Keikhaei [32]	Hb × RDW × 100/ RBC^2^ × MCHC	< 21	2010
19	Nishad(Thal) [33]	0.615 MCV + 0.518 MCH + 0.446 RDW	< 59	2012
20	Wongprachum [34]	(MCV × RDW/RBC) – 10 HB	> 104	2012
21	Sehgal [15]	MCV2 /RBC	< 972	2013
22	Sargolzaie [35]	125.643 + 44.304 × RBC-20.932 × Hb-2.501 × MCV + 20.302 × MCH-12.183 × MCHC	< 0.5	2014
23	Pornprasert [36]	MCHC	< 31	2014
24	Sirachainan [37]	1.5 HB – 0.05 MCV	> 14	2014
25	Plengsuree [38]	RDW/RBC	< 3.3	2015
26	Bordbar [11]	│80-MCV│ × │27-MCH│	> 44.76	2015
27	Hisham [18]	MCH × RDW / RBC	< 67	2015
28	Hameed [18]	MCH × HCT × RDW / (RBC × Hb)2	< 220	2015
29	Chandra [13]	RBC × MCHC × MPV / RDW × PLT	> 0.22	2016
30	Matos and Carvalho(M&C) [39]	1.91 × RBC + 0.44 × MCHC	> 23.85	2016
31	Ravanbakhsh -F1 [40]	MCV / HCT	< 2.0	2016
32	Ravanbakhsh-F2 [40]	RDW − 3 × RBC	< 1.5	2016
33	Ravanbakhsh-F3 [40]	MCV × RDW – (100 × RBC)	< 600	2016
34	Ravanbakhsh-F4 [40]	MCV × Hb / RDW × RBC	< 10	2016
35	Zaghloul-1 [41]	Hb + HCT + RBC	> 52.5	2016
36	Zaghloul-2 [41]	Hb + HCT + RBC − RDW	> 37.1	2016
37	Kandhro-1 [21]	RBC / HCT + 0.5 × RDW	< 8.2	2017
38	Kandhro-2 [21]	RDW × 5 / RBC	< 16.8	2017
39	Merdin-1 [42]	RDW × RBC / MCV	> 1.27	2018
40	Merdin-2 [42]	RDW × RBC × Hb / MCV	> 14.7	2018
41	Alparslan [42]	10log (MCH × MCHC × RDW / RBC)	< 3.34	2018
42	Roth (SVM) [43]	1.45 × (MCV − 82.8) / 10.28 + 0.66 × (MCH − 27.0) / 3.9 + 0.98	< 0.0	2018
43	Cruise [44]	MCHC + 0.603 RBC + 0.523 RDW	≥ 42.63	2019
44	XS-1	Combination of log(e) from 3 indices	< 4	2022

**Table 3:** Differential formulas ROC for 44 thalassemias and iron deficiency anemia

DF	AUC(95%CI)	Optimal cut-off	Original cut-off	TP	FP	FN	TN	Accuracy(%)	Sensitivity(%)	Specificity(%)	Youden Index(%)
E&F	0.67(0.617–0.719)	-31.930	< 0	228	200	1	1	53.26	99.56	0.50	0.00
RBC	0.84(0.805–0.88)	4.545	> 5	81	3	148	198	64.88	35.37	98.51	0.34
Mentzer	0.91(0.878–0.935)^*^	18.227	< 13	62	0	167	201	61.16	27.07	100.00	0.27
Srivastava	0.92(0.89–0.943)^*^	5.915	< 3.8	26	0	203	201	52.79	11.35	100.00	0.11
S&L	0.90(0.874–0.934)^*^	1,820.666	< 1530	167	13	62	188	82.56	72.93	93.53	0.66
Bessman	0.75(0.704–0.801)	13.650	< 15	131	169	98	32	37.91	57.21	15.92	-0.27
Ricerca	0.60(0.551–0.658)	2.939	< 3.3	171	130	58	71	56.28	74.67	35.32	0.10
G&K	0.83(0.786–0.866)	75.777	< 65	84	4	145	197	65.35	36.68	98.01	0.35
Das.Gupta	0.75(0.7–0.793)	0.498	> 0	193	124	36	77	62.79	84.28	38.31	0.23
RDW.index	0.87(0.84–0.909)	252.550	< 220	117	5	112	196	72.79	51.09	97.51	0.49
TI-MCHD	0.86(0.825–0.899)	0.329	< 0.34	222	145	7	56	64.65	96.94	27.86	0.25
TI-MDHL	0.76(0.718–0.808)	1.510	> 1.5	135	39	94	162	69.07	58.95	80.60	0.40
Huber.-Herklotz	0.64(0.587–0.691)	22.041	< 20	23	8	206	193	50.23	10.04	96.02	0.06
Sirdah	0.90(0.867–0.928)^*^	40.945	< 27	36	0	193	201	55.12	15.72	100.00	0.16
Kerman.I	0.92(0.89–0.944)^*^	473.980	< 300	94	0	135	201	68.60	41.05	100.00	0.41
Kerman.II	0.90(0.873–0.932)^*^	45.788	< 85	228	199	1	2	53.49	99.56	1.00	0.01
Ehsani	0.91(0.882–0.938)^*^	37.500	< 15	63	0	166	201	61.40	27.51	100.00	0.28
Keikhaei	0.87(0.839–0.90)	25.695	< 21	89	3	140	198	66.74	38.86	98.51	0.37
Nishad	0.91(0.88–0.939)^*^	71.604	< 59	81	0	148	201	65.58	35.37	100.00	0.35
Wongprachum	0.81(0.763–0.847)	127.029	> 104	132	187	97	14	33.95	57.64	6.97	-0.35
Sehgal	0.91(0.883–0.939)^*^	1,421.514	< 972	103	0	126	201	70.70	44.98	100.00	0.45
Sargolzaie	0.89(0.853–0.918)	11.274	< 0.5	57	3	172	198	59.30	24.89	98.51	0.23
Pornprasert	0.86(0.825–0.899)	32.850	< 31	23	5	206	196	50.93	10.04	97.51	0.08
Sirachainan	0.74(0.688–0.785)	166.220	> 14	229	201	0	0	53.26	100.00	0.00	0.00
Plengsuree	0.60(0.551–0.658)	2.939	< 3.3	171	130	58	71	56.28	74.67	35.32	0.10
Bordbar	0.70(0.648–0.749)	64.880	> 44.76	135	47	94	154	67.21	58.95	76.62	0.36
Hisham	0.90(0.873–0.933)*	81.779	< 67	96	0	133	201	69.07	41.92	100.00	0.42
Hameed	0.77(0.721–0.811)	49.905	< 220	229	201	0	0	53.26	100.00	0.00	0.00
Chandra	0.52(0.469–0.579)	0.590	> 0.22	227	198	2	3	53.49	99.13	1.49	0.01
M&C	0.68(0.632–0.733)	23.169	> 23.85	58	10	171	191	57.91	25.33	95.02	0.20
Ravanbakhsh F1	0.84(0.805–0.88)	2.171	< 2.0	83	3	146	198	65.35	36.24	98.51	0.35
Ravanbakhsh F2	0.60(0.544–0.651)	-0.295	< 1.5	172	142	57	59	53.72	75.11	29.35	0.04
Ravanbakhsh F3	0.86(0.823–0.895)	673.350	< 600	138	13	91	188	75.81	60.26	93.53	0.54
Ravanbakhsh F4	0.87(0.839–0.908)	13.714	< 10	114	8	115	193	71.40	49.78	96.02	0.46
Zaghloul.1	0.72(0.67–0.768)	153.530	> 52.5	229	201	0	0	53.26	100.00	0.00	0.00
Zaghloul.2	0.73(0.678–0.776)	139.120	> 37.1	229	201	0	0	53.26	100.00	0.00	0.00
Kandhro.1	0.76(0.709–0.805)	6.971	< 8.2	186	179	43	22	48.37	81.22	10.95	-0.08
Kandhro.2	0.60(0.551–0.658)	14.696	< 16.8	176	135	53	66	56.28	76.86	32.84	0.10
Merdin.1	0.88(0.843–0.909)	0.709	> 1.27	41	2	188	199	55.81	17.90	99.00	0.17
Merdin.2	0.83(0.792–0.869)	93.715	> 14.7	229	201	0	0	53.26	100.00	0.00	0.00
Alparslan	0.92(0.894–0.947)^*^	4.381	< 3.34	102	0	127	201	70.47	44.54	100.00	0.45
Roth..SVM	0.90(0.873–0.933)^*^	0.911	< 0.0	167	14	62	187	82.33	72.93	93.03	0.66
Cruise	0.86(0.823–0.897)	339.503	≥ 42.63	229	201	0	0	53.26	100.00	0.00	0.00
XS-1	0.92(0.888–0.943)^*^	3.989	< 4	188	22	41	179	85.35	82.10	89.05	0.71

### Web-based tool implementation

**Fig. 2:**
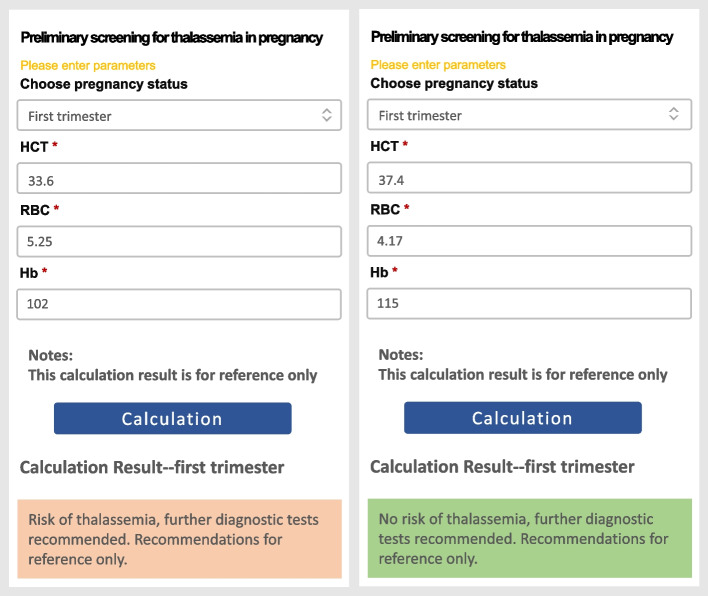
Screenshots of the web-based tool after submission of laboratory data, which is available at http://yyy.yiyiy.top:28992/XS-1/

An accessible Web-based tool was established with the XS-1 model to help the medical team quickly determine whether pregnant women have the characteristics of thalassemia or not during the outpatient phase (Fig. 2). Enter the Hct, RBC and Hb data of the pregnant woman, click the button to start the calculation process, and then the prediction result will be displayed in the interface.

## Discussion

TT and IDA are two recognized causes of two prevalent microcytic anemias in pregnant women, and these two hematologic disorders are comparable in different clinical and research contexts. Physiological changes during pregnancy exacerbate the severity of anemia and are significantly associated with the increased risk of pregnancy outcomes. Early identification of TT and IDA among pregnant women is important for genetic counseling and appropriate therapeutic intervention. Misdiagnosis of TT has an impact on possible homozygous offsprings. Women with TT and ferritin less than 20, should be given iron replacement therapy and if the ferritin remains less that < 20 a month after oral treatment they should be treated with an iron infusion. Currently, the screening method for TT is based on an increase in HbA2, while the diagnosis of IDA is based on an increase in Total Iron binding capacity (TIBC) and a decrease in serum ferritin (SF). Since 1973, different mathematical formulas using only complete blood counts have been consistently proposed to identify TT and IDA. These formulas have shown different clinical utility, but no single formula is recommended in the guidelines, nor are they confirmed in pregnant women.

Previous studies often selected data from patients with microcytic hypochromic anemia to construct or validate formulas [45], and there were two misunderstandings: the first was to ignore the group of TT combined with IDA [30, 46]; the other was ignoring the group with TT or ID without anemia. However, anemia during pregnancy develops progressively, which means anemia may not occur in the first trimester but the second and later stages of pregnancy. In addition, human bodies may adapt to environmental changes. With the changes in hormone levels during pregnancy, the increase in blood volume leads to an increase in Hb dilution [47]. The mother will achieve a new dynamic balance through self-regulation. The consistent and uninterrupted division of various blood cells generates new cells all the time and divides immediately. The red blood cells, as a natural object, has a growth process satisfies the natural growth limit, that is, the growth per unit time can expand at most e times of the natural constant.This is an adaptive process in the human body, and in order to ensure the normal operation of the human body, the number of RBC is increased to carry enough oxygen and implement other functions, so we used the logarithm algorithm, also based on this natural law. During pregnancy, the body responds to a progressive decrease in dilutive hemoglobin by gradually increasing the number of red blood cells or changing their size to achieve a new balance. By combining hematological parameters based on natural logarithms, the maximum difference of erythrocyte increase limit between TT and IDA can be found, so as to construct the discrimination formula. Furthermore, the diagnosis of TT was made based on genetic analysis in our study. Most of the discriminant formulas described in previous literature are individually formulated for diagnosis based on genetic testing. This may be an important factor contributing to the difference in diagnostic performance between our results and others.

In addition, many formulas have been clarified for the scope of usage since being published. For example, the England and Fraser formula is used in people suspected of having microcytosis, and is of little significance to people with normal MCV [17]. Formulas like England and Fraser [17], Mentzer [23], Srivastava [24], Shine and Lal [25], and Ehsani [16] are not suitable for the identification of anemia due to bleeding, hemolysis or pregnancy. Some formulas have other requirements, such as the Huber–Herklotz [29] formula for people with MCV < 73, the Kerman II [14] and Nishad [33] formulas for people with MCV < 80, the Wongprachum [34] formula for vegetarians, and the Pornpraser [36] and Sirachainan [37] formulas for students or children. Although Roth (SVM) [43] formula is suitable for fertile women, it cannot discriminate iron-deficiency anemia in the original study, and it was slightly inferior to the newer formula XS-1 in this study.

Other discrimination formulas have included Ret% [48]. Since Ret% exam has not been popularized in China, we did not include such formulas. In this study, the group of TT combined with IDA was excluded, and pregnant women with Hb > 110 g/L were included, so the performance of most formulas was relatively low, which is consistent with Keikhaei's [32] research, and a certain sensitivity can also be achieved by changing the critical value of the formula and specificity [21].

Clinical empirical research was performed on the 43 proposed formulas with data from 430 pregnant women, and comparison was made with the newly introduced XS-1 formula. An ideal formula for identifying thalassemia should have very high sensitivity and relatively good specificity to detect the largest number of TT patients while excluding IDA patients. Among the 44 formulas, only 13 formulas had AUC > 0.9. The sensitivity and specificity of the newly introduced formula XS-1 were 82.10 and 89.05 with the Youden Index ranking first, showing the new formula better than the general discriminant formula. Except for Shine and Lal and Roth (SVM) formulas, the discriminative abilities of other formulas in this study are lower in this study than in the original results.

Although the 10 hematological parameters of TT and IDA in this study were statistically different, considering the current laboratory blood cell detection methods, only RBC, Hb, and HCT are the measurement results, while MCV, MCH, MCHC, RDW-CV are calculated from the previous values. And some studies have found that MCV does not accurately reflect erythropoiesis [49]. In any progressive iron depletion, the latest red blood cell volume is the smallest, while thalassemia is not iron deficient, so its red blood cell MCV does not change It has also been found that MCV is not a good indicator to distinguish TT from IDA [50]. Therefore, in this study, only three indices: RBC, Hb, and HCT, were selected to be presented on the primary screening webpage. It is suggested that the source and calculation method of hematological parameters should be considered in future research.

Limitations of our study include the small number of pregnant women with TT and IDA, limited to the first trimester and single-centered data. Furthermore, we did not construct a validation group to varify, and more clinical studies should be conducted in different stage of pregnancy to evaluate the performance of the XS-1 formula.

## Conclusion

TT and IDA can be discriminated without using expensive tests with high-performance metrics. We proposed a new discriminant formula XS-1 for screening TT and IDA and compared its performance with 43 published formulas. The results show that no single discriminant formula can provide 100% sensitivity and specificity. XS-1 is superior to all published formulas, and can be used for discrimination of TT and IDA in the first trimester. Whether they appear to be iron deficient or not, pregnant women should be screened for TT.

## Data Availability

The datasets used and/or analysed during the current study available from the corresponding author on reasonable request or available from Xiao Shuang.

## Abbreviations

TT: Thalassemia Trait
IDA: Iron deficiency anemia
ID: Iron deficiency
MHA: Microcytic hypochromic anemia
SF: Serum ferritin
TIBC: Total iron binding capacity
WBC: White blood cell count
RBC: Red blood cell
Hb: Hemoglobin
HCT: Hematocrit
MCV: Mean corpuscular volume
MCH: Mean corpuscular hemoglobin
MCHC: Mean corpuscular hemoglobin concentration
RDW: Red blood cell volume distribution width
PLT: Platelet count/blood platelet count
DF: Discriminant formula
ROC: Receiver operating characteristic curve

## References

[1] Daru J, Zamora J, Fernández-Félix BM, Vogel J, Oladapo OT, Morisaki N (2018). Risk of maternal mortality in women with severe anaemia during pregnancy and post partum: a multilevel analysis. Lancet Glob Health.

[2] McLean E, Cogswell M, Egli I, Wojdyla D, De Benoist B (2009). Worldwide prevalence of anaemia, WHO vitamin and mineral nutrition information system, 1993–2005. Public Health Nutr.

[3] Higgs DR, Engel JD, Stamatoyannopoulos G (2012). Thalassaemia. The lancet.

[4] Yin A, Li B, Luo M, Xu L, Wu L, Zhang L (2014). The prevalence and molecular spectrum of α-and β-globin gene mutations in 14,332 families of Guangdong Province, China. PLoS ONE.

[5] Xiong F, Sun M, Zhang X, Cai R, Zhou Y, Lou J (2010). Molecular epidemiological survey of haemoglobinopathies in the Guangxi Zhuang Autonomous Region of southern China. Clin Genet.

[6] Obstetrics Subgroup CSoO, Gynecology CMA. [Guideline of preconception and prenatal care(2018)]. Zhonghua Fu Chan Ke Za Zhi. 2018;53(1):7–13. Epub 2018/01/30. 10.3760/cma.j.issn.0529-567X.2018.01.003. PubMed PMID: 29374879. 10.3760/cma.j.issn.0529-567X.2018.01.003 29374879

[7] Tsamesidis I, Fozza C, Vagdatli E (2017). Total antioxidant capacity in Mediterranean β-thalassemic patients[J]. Advances in Clinical and Experimental Medicine.

[8] Vafaei H, Karimi S, Akbarzadeh Jahromi M, Asadi N, Kasraeian M. The effect of mother's beta-thalassemia minor on placental histology and neonatal outcomes. J Matern Fetal Neonatal Med. 2020:1–8. Epub 2020/06/05. 10.1080/14767058.2020.1774540. PubMed PMID: 32495664. 10.1080/14767058.2020.1774540 32495664

[9] Adler A, Wainstock T, Sheiner E (2021). Prenatal exposure to maternal beta-thalassemia minor and the risk for long-term hematologic morbidity in the offspring: a population-based cohort study. Early Hum Dev.

[10] Bessman JD, Feinstein DI (1979). Quantitative anisocytosis as a discriminant between iron deficiency and thalassemia minor. Blood.

[11] Bordbar E, Taghipour M, Zucconi BE (2015). Reliability of different RBC indices and formulas in discriminating between β-thalassemia minor and other microcytic hypochromic cases. Mediterr J Hematol Infect Dis.

[12] Burdick CO, Ntaios G, Rathod D (2009). Separating thalassemia trait and iron deficiency by simple inspection. Am J Clin Pathol.

[13] Chandra H, Shrivastava V, Chandra S, Rawat A, Nautiyal R (2016). Evaluation of platelet and red blood cell parameters with proposal of modified score as discriminating guide for iron deficiency anemia and β-thalassemia minor. J Clin Diagn Res.

[14] Cohan N, Ramzi M (2008). Evaluation of sensitivity and specificity of Kerman index I and II in screening beta thalassemia minor. Scientific Journal of Iran Blood Transfus Organ.

[15] Dharmani P, Sehgal K, Dadu T, Mankeshwar R, Shaikh A, Khodaiji S (2013). Developing a new index and its comparison with other cbc-based indices for screening of beta thalassemia trait in a tertiary care hospital: 704. Int J Lab Hematol.

[16] Ehsani M, Shahgholi E, Rahiminejad M, Seighali F, Rashidi A (2009). A new index for discrimination between iron deficiency anemia and beta-thalassemia minor: results in 284 patients. Pakistan journal of biological sciences: PJBS.

[17] England J, Fraser P (1973). Differentiation of iron deficiency from thalassaemia trait by routine blood-count. The Lancet.

[18] Getta HA, Yassen H, Said HM. Hi & Ha, are new indices in differentiation between iron deficiency anemia and beta-thalassaemia trait. a study in Sulaimani City-Kurdistan, Iraq. J Dental Med Sci. 2015;14:67–72.

[19] Green R, King R (1989). A new red cell discriminant incorporating volume dispersion for differentiating iron deficiency anemia from thalassemia minor. Blood Cells.

[20] Gupta AD, Hegde C, Mistri R (1994). Red cell distribution width as a measure of severity of iron deficiency in iron deficiency anaemia. Indian J Med Res.

[21] Hafeez Kandhro A, Shoombuatong W, Prachayasittikul V, Nuchnoi P (2017). New bioinformatics-based discrimination formulas for differentiation of thalassemia traits from iron deficiency anemia. Laboratory medicine.

[22] Chinese Society of Perinatal Medicine (2014). Guidelines for diagnosis and treatment of iron deficiency and iron deficiency anemia in pregnancy. Chinese Journal of Perinatal Medicine.

[23] Mentzer W (1973). Differentiation of iron deficiency from thalassaemia trait. The lancet.

[24] Schriever H, Srivastava P (1973). Differentiation of thalassaemia minor from iron deficiency. The Lancet.

[25] Shine I, Lal S (1977). A strategy to detect β-thalassaemia minor. The Lancet.

[26] Ricerca B, Storti S, d'Onofrio G, Mancini S, Vittori M, Campisi S (1987). Differentiation of iron deficiency from thalassaemia trait: a new approach. Haematologica.

[27] Jayabose S, Giamelli J, LevondogluTugal O, Sandoval C, Ozkaynak F, Visintainer P (1999). # 262 Differentiating iron deficiency anemia from thalassemia minor by using an RDW-based index. J Pediatr Hematol Oncol.

[28] Telmissani OA, Khalil S, Roberts GT (1999). Mean density of hemoglobin per liter of blood: a new hematologic parameter with an inherent discriminant function. Lab Hematol.

[29] Huber A, Ottiger C, Risch L, Regenass S, Hergersberg M, Herklotz R (2004). Thalassämie-Syndrome: Klinik und diagnose syndromes thalassémiques: clinique et diagnostic. Schweiz Med Forum.

[30] Sirdah M, Tarazi I, Al Najjar E, Al HR (2008). Evaluation of the diagnostic reliability of different RBC indices and formulas in the differentiation of the β-thalassaemia minor from iron deficiency in Palestinian population. Int J Lab Hematol.

[31] Ehsani M, Darvish A, Aslani A, Seighali F (2005). A new formula for differentiation of iron deficiency anemia (IDA) and thalassemia trait (TT). Turk J Haematol (Supp).

[32] Keikhaei B (2010). A new valid formula in differentiating iron deficiency anemia from ß-thalassemia trait. Pakist J Med Sci.

[33] Nishad AAN, Pathmeswaran A, Wickremasinghe A, Premawardhena A. The Thal-index with the BTT prediction. exe to discriminate ß-thalassaemia traits from other microcytic anaemias. Thalassemia Reports. 2012.

[34] Wongprachum K, Sanchaisuriya K, Sanchaisuriya P, Siridamrongvattana S, Manpeun S, Schlep FP (2012). Proxy indicators for identifying iron deficiency among anemic vegetarians in an area prevalent for thalassemia and hemoglobinopathies. Acta Haematol.

[35] Sargolzaie N, Miri-Moghaddam E (2014). A local equation for differential diagnosis of β-thalassemia trait and iron deficiency anemia by logistic regression analysis in Southeast Iran. Hemoglobin.

[36] Pornprasert S, Panya A, Punyamung M, Yanola J, Kongpan C (2014). Red cell indices and formulas used in differentiation of β-thalassemia trait from iron deficiency in Thai school children. Hemoglobin.

[37] Sirachainan N, Iamsirirak P, Charoenkwan P, Kadegasem P, Wongwerawattanakoon P, Sasanakul W (2014). New mathematical formula for differentiating thalassemia trait and iron deficiency anemia in thalassemia prevalent area: a study in healthy school-age children. Southeast Asian J Trop Med Public Health.

[38] Plengsuree S, Punyamung M, Yanola J, Nanta S, Jaiping K, Maneewong K (2015). Red cell indices and formulas used in differentiation of β-thalassemia trait from iron deficiency in Thai adults. Hemoglobin.

[39] Matos JF, Dusse  L, Borges KB , de Castro  RL, Coura-Vital W, Carvalho  MdG (2016). A new index to discriminate between iron deficiency anemia and thalassemia trait. Rev Bras Hematol Hemoter.

[40] Ravanbakhsh M, Mousavi SA (2016). Diagnostic reliability check of red cell indices in differentiating iron deficiency anemia (IDA) from beta thalassemia minor (BTT). Hormozgan Medical Journal.

[41] Zaghloul A, Al-Bukhari T, Bajuaifer N, Shalaby M, Al-Pakistani H, Halawani SH (2016). Introduction of new formulas and evaluation of the previous red blood cell indices and formulas in the differentiation between beta thalassemia trait and iron deficiency anemia in the Makkah region. Hematology.

[42] Merdin A (2018). Suggestion of new formulae to be used in distinguishing beta thalasemia trait from iron deficiency anemia. Acta Med Mediter.

[43] Roth IL, Lachover B, Koren G, Levin C, Zalman L, Koren A (2018). Detection of β-thalassemia carriers by red cell parameters obtained from automatic counters using mathematical formulas. Mediterr J Hematol Infect Dis.

[44] Jahangiri M, Rahim F, Malehi AS (2019). Diagnostic performance of hematological discrimination indices to discriminate between betaeta thalassemia trait and iron deficiency anemia and using cluster analysis: introducing two new indices tested in Iranian population. Sci Rep.

[45] Hoffmann JJ, Urrechaga E, Aguirre U (2015). Discriminant indices for distinguishing thalassemia and iron deficiency in patients with microcytic anemia: a meta-analysis. Clin Chem Lab Med.

[46] Aslan D, Değermenci Ş (2022). peripheral blood erythrocyte parameters in Β-Thalassemia minor with coexistent iron deficiency: comparisons between iron-deficient and-sufficient carriers. Thalassemia Reports.

[47] Koller O (1982). The clinical significance of hemodilution during pregnancy. Obstet Gynecol Surv.

[48] Xiao H, Wang Y, Ye Y, Yang C, Wu X, Wu X (2021). Differential diagnosis of thalassemia and iron deficiency anemia in pregnant women using new formulas from multidimensional analysis of red blood cells. Ann Transl Med.

[49] Ambayya A, Sahibon S, Yang TW, Zhang Q-Y, Hassan R, Sathar J (2021). A novel algorithm using cell population data (VCS Parameters) as a screening discriminant between alpha and beta thalassemia traits. Diagnostics.

[50] Rashwan  NI, Ahmed  AE-A, Hassan  MH, Mohammed ME, Bakri  AH (2022). Hematological indices in differentiation between iron deficiency anemia and beta-thalassemia trait. Int J Pediatr.

